# Phosphoproteomics of Aspergillus fumigatus Exposed to the Antifungal Drug Caspofungin

**DOI:** 10.1128/mSphere.00365-20

**Published:** 2020-05-27

**Authors:** Eliciane Cevolani Mattos, Giuseppe Palmisano, Gustavo H. Goldman

**Affiliations:** a Faculdade de Ciências Farmacêuticas de Ribeirão Preto, Universidade de São Paulo, Ribeirão Preto, Brazil; b Departamento de Parasitologia, Instituto de Ciências Biomédicas, Universidade de São Paulo, São Paulo, Brazil; University of Georgia

**Keywords:** *Aspergillus fumigatus*, caspofungin, phosphoproteomics, MAP kinases, transcription factors

## Abstract

Aspergillus fumigatus is an opportunistic and allergenic pathogenic fungus, responsible for fungal infections in humans. A. fumigatus infections are usually treated with polyenes, azoles, or echinocandins. Echinocandins, such as caspofungin, can inhibit the biosynthesis of the β-1,3-glucan polysaccharide, affecting the integrity of the cell wall and leading to fungal death. In some A. fumigatus strains, caspofungin treatment at high concentrations induces an increase of fungal growth, a phenomenon called the caspofungin paradoxical effect (CPE). Here, we analyze the proteome and phosphoproteome of the A. fumigatus wild-type strain and of mitogen-activated protein kinase (MAPK) *mpkA* and *sakA* null mutant strains during CPE (2 μg/ml caspofungin for 1 h). The wild-type proteome showed 75 proteins and 814 phosphopeptides (corresponding to 520 proteins) altered in abundance in response to caspofungin treatment. The Δ*mpkA* (Δ*mpkA* caspofungin/wild-type caspofungin) and Δ*sakA* (Δ*sakA* caspofungin/wild-type caspofungin) strains displayed 626 proteins and 1,236 phosphopeptides (corresponding to 703 proteins) and 101 proteins and 1,217 phosphopeptides (corresponding to 645 proteins), respectively, altered in abundance. Functional characterization of the phosphopeptides from the wild-type strain exposed to caspofungin showed enrichment for transcription factors, protein kinases, and cytoskeleton proteins. Proteomic analysis of the Δ*mpkA* and Δ*sakA* mutants indicated that control of proteins involved in metabolism, such as in production of secondary metabolites, was highly represented in both mutants. Results of functional categorization of phosphopeptides from both mutants were very similar and showed a high number of proteins with decreased phosphorylation of proteins involved in transcriptional control, DNA/RNA binding, cell cycle control, and DNA processing. This report reveals novel transcription factors involved in caspofungin tolerance.

**IMPORTANCE** Aspergillus fumigatus is an opportunistic human-pathogenic fungus causing allergic reactions or systemic infections, such as invasive pulmonary aspergillosis in immunocompromised patients. Caspofungin is an echinocandin that impacts the construction of the fungal cell wall by inhibiting the biosynthesis of the β-1,3-glucan polysaccharide. Caspofungin is a fungistatic drug and is recommended as a second-line therapy for treatment of aspergillosis. Treatment at high concentrations induces an increase of fungal growth, a phenomenon called the caspofungin paradoxical effect (CPE). Collaboration between the mitogen-activated protein kinases (MAPK) of the cell wall integrity (MapkA) and high-osmolarity glycerol (SakA) pathways is essential for CPE. Here, we investigate the global proteome and phosphoproteome of A. fumigatus wild-type, Δ*mpkA*, and Δ*sakA* strains upon CPE. This study showed intense cross talk between the two MAPKs for the CPE and identified novel protein kinases and transcription factors possibly important for CPE. Increased understanding of how the modulation of protein phosphorylation may affect the fungal growth in the presence of caspofungin represents an important step in the development of new strategies and methods to combat the fungus inside the host.

## Abstract (precis)

Aspergillus fumigatus is an opportunistic human-pathogenic fungus causing allergic reactions or systemic infections, such as invasive pulmonary aspergillosis in immunocompromised patients. Caspofungin is an echinocandin that impacts the construction of the fungal cell wall by inhibiting the biosynthesis of the β-1,3-glucan polysaccharide. Caspofungin is a fungistatic drug and is recommended as a second-line therapy for treatment of aspergillosis. Treatment at high concentrations induces an increase of fungal growth, a phenomenon called the caspofungin paradoxical effect (CPE). Collaboration between the mitogen-activated protein kinases (MAPK) of the cell wall integrity (MapkA) and high-osmolarity glycerol (SakA) pathways is essential for CPE. Here, we investigate the global proteome and phosphoproteome of A. fumigatus wild-type, Δ*mpkA*, and Δ*sakA* strains upon CPE. This study showed intense cross talk between the two MAPKs for the CPE and identified novel protein kinases and transcription factors possibly important for CPE. Increased understanding of how the modulation of protein phosphorylation may affect the fungal growth in the presence of caspofungin represents an important step in the development of new strategies and methods to combat the fungus inside the host.

## INTRODUCTION

Aspergillus fumigatus is an opportunistic and allergenic pathogenic fungus that is responsible for a high incidence of fungal infections in humans and several pathologies in immunocompromised individuals. Fungal infections, such as invasive aspergillosis (IA), are usually treated with polyenes, azoles, or echinocandins (1). Polyenes are fungicidal and act by disruption of the fungal cell membrane by physically binding to the membrane ergosterol, resulting in pore formation and cell death (1). Azoles affect the biosynthesis of ergosterol, causing damage to the fungal membrane and resulting in cell death by membrane lysis or inhibition of fungal growth (1). Echinocandins can inhibit the biosynthesis of the β-1,3-glucan polysaccharide, the major component of the fungal cell wall, affecting the integrity of the cell wall and leading to fungal death (1, 2). Triazoles (voriconazole, for example) have fungistatic activities and are applied as a primary treatment in invasive aspergillosis therapy, whereas echinocandins (such as caspofungin [caspo], anidulafungin, and micafungin) act as fungistatic agents and are used as a second-line drug treatment in patients with infections that are recalcitrant to the primary treatments (1–3). The emergence of A. fumigatus clinical isolates resistant to azoles and echinocandins has been considered a potential public health issue (4).

In some A. fumigatus strains, tolerance to caspofungin at high concentrations induces an increase in fungal growth, a phenomenon called the caspofungin paradoxical effect (CPE) (5, 6). Compensatory reactions such as the transcriptional stimulation of genes encoding chitin synthases and the subsequent increase of chitin content in the cell wall are due to the activation of stress-activated signaling pathways (4, 6). High caspofungin concentrations elicit a spike in cytosolic calcium, which activates calcineurin and CrzA to promote the CPE (7, 8). Upon promotion of the CPE, calcineurin dephosphorylates the transcription factor (TF) CrzA, which is translocated to the nucleus and activates the expression of chitin synthases (9). A novel basic leucine zipper ZipD transcription factor was identified to function in the calcium-calcineurin pathway and was involved in the CPE (9). Recently, we identified four transcription factors whose null mutants are susceptible to calcium and are also involved in CPE (10).

CPE is also dependent on all four A. fumigatus mitogen-activated protein kinases (MAPKs). The cell wall integrity (CWI) pathway involving the MAPK MpkA (2) activates the RlmA transcription factor, which regulates the expression of chitin synthase genes in response to different concentrations of caspofungin (9, 11, 12). A. fumigatus MAPKs, including high-osmolarity glycerol (HOG) MAPK SakA and MpkC, are also involved in CPE, activating TFs AtfA to AtfD, which are important for CPE maintenance (13, 14). The null mutant for the MAPK MpkB, homologous to yeast Fus3 (15), is more susceptible to caspofungin and had also lost the CPE (15). A cross talk interaction between SakA and MpkA has already been observed for the adaptation to caspofungin (13, 16, 17).

It has been demonstrated that A. fumigatus paradoxically growing hyphae emerging from microcolonies are initially devoid of β-1,3-glucan (18) but, intriguingly, that these hyphae expose β-1,3-glucan in later growth stages (18). Fks1 glucan synthase, the main target of caspofungin, relocates to the hyphal tips during these later growth stages, suggesting that β-1,3-glucan synthase activity is restored (18, 19). Interestingly, a novel mechanism has been proposed to explain this increased glucan accumulation and Fks1 relocation to the hyphal tips (20). Analysis of the lipid microenvironment of an Fks1 from an A. fumigatus caspofungin-resistant mutant showed an increase in the abundance of dihydrosphingosine (DhSph) and phytosphingosine (PhSph), suggesting that caspofungin induced an alteration in the composition of plasma membrane lipids surrounding Fks1 glucan synthase, rendering it resistant to echinocandins (20).

Genetic or chemical inhibition of heat shock protein 90 (Hsp90) abolished the CPE (21). Besides Hsp90, another molecular chaperone, Hsp70, is also important for the regulation of CPE together with their cochaperone Hop/StiA (22). Transcriptional profiling has shown that the main effect of reducing the Hsp90 effect is the reduction of expression of the genes of the mitochondrial respiratory chain, in particular, that of the genes encoding NADH-ubiquinone oxidoreductases (complex I) (23, 24).

The members of our laboratory are interested in understanding which genetic determinants are involved in A. fumigatus CPE. Recently, we evaluated the global A. fumigatus phosphoproteome-exposed cell wall damage mediated by Congo red (CR), identifying 485 proteins putatively involved in the cell wall stress response (25). Among these proteins, we have isolated five novel transcription factors (TFs) that were phosphorylated upon exposure to CR. The TF null mutants for three of these genes were more susceptible to CR, calcofluor white (CFW), and caspofungin with reduced CPE (25). Here, we extended these studies by analyzing the proteome and phosphoproteome of the A. fumigatus wild-type (WT) and Δ*mpkA* and Δ*sakA* mutant strains during the stress with caspofungin.

## RESULTS

### Proteome and phosphoproteome analysis of A. fumigatus upon caspofungin stress.

The study of proteins and their posttranslational modifications in an organism under some stress conditions provided an important insight into the biological processes and signaling pathways modulated during the environmental response. Previously, we had observed that fungal mycelia grown in liquid cultures are more susceptible to high concentrations of caspofungin, and in agreement, the CPE of A. fumigatus strain CEA17 was already observed at 2 μg/ml caspofungin in liquid minimal medium (MM) for 1 h, whereas the onset of the CPE was observed at 8 μg/ml on solid medium (9). To understand how A. fumigatus reacts to CPE concentrations and about the involvement of MAPKs in that response, we perform proteomic and phosphoproteomic analysis of the wild-type, Δ*sakA*, and Δ*mpkA* strains left untreated (control) or treated with 2 μg/ml caspofungin for 1 h.

**FIG 1:**
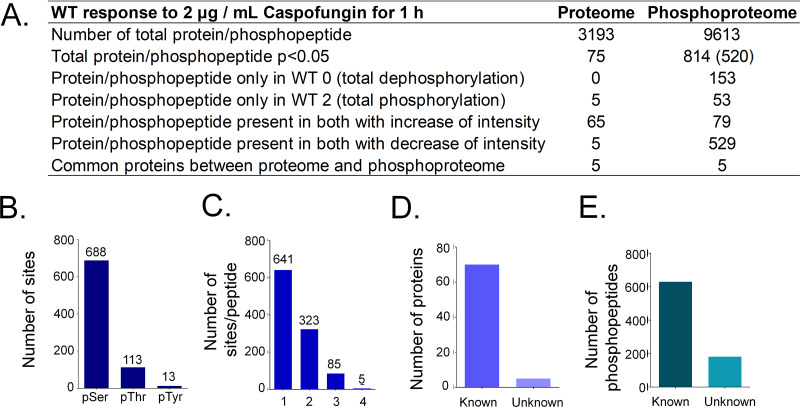
Proteome and phosphoproteome of A. fumigatus wild-type strain exposed to caspofungin. (A) Summary table of all proteins and phosphopeptides modulated in wild-type strain upon exposure to caspofungin. (B) Number of phosphorylated residues of serine, threonine, or tyrosine identified. (C) Number of sites of phosphorylation per peptide. (D) Number of proteins. (E) Number of known and unknown proteins.

To understand the signaling pathways and identify new targets that may be involved in the fungal response to caspofungin, we started our study by the analysis of the wild-type response to the drug. The analysis of total proteins identified 3,193 unique proteins. The wild-type proteome showed that 75 proteins were altered in abundance in response to 2 μg/ml caspofungin treatment, with most of them showing increased total levels (70 proteins with increased versus 5 proteins with decreased total levels) (Fig. 1A; see also Table S1A at https://doi.org/10.6084/m9.figshare.12315212). The results obtained from samples enriched with TiO_2_ enrichment for phosphorylated proteins showed a total of 9,613 phosphopeptides, with 6,522 with localization probability levels higher than 75% (class I phosphosites) (Fig. 1A; see also Table S1B). As determined using Student's *t* test, 814 phosphopeptides were differentially expressed between caspofungin-treated wild-type strain and the control (nontreated [0]), with most of them (682 [83.7%]) being dephosphorylated or decreasing their phosphorylation levels (Fig. 1A; see also Table S1B). The analysis of the sites of phosphorylation indicated the presence of 688 residues of serine, 113 residues of threonine, and 13 residues of tyrosine (Fig. 1B) and indicated 1 (641) or 2 (323) phosphosites per peptide (Fig. 1C).

**FIG 2:**
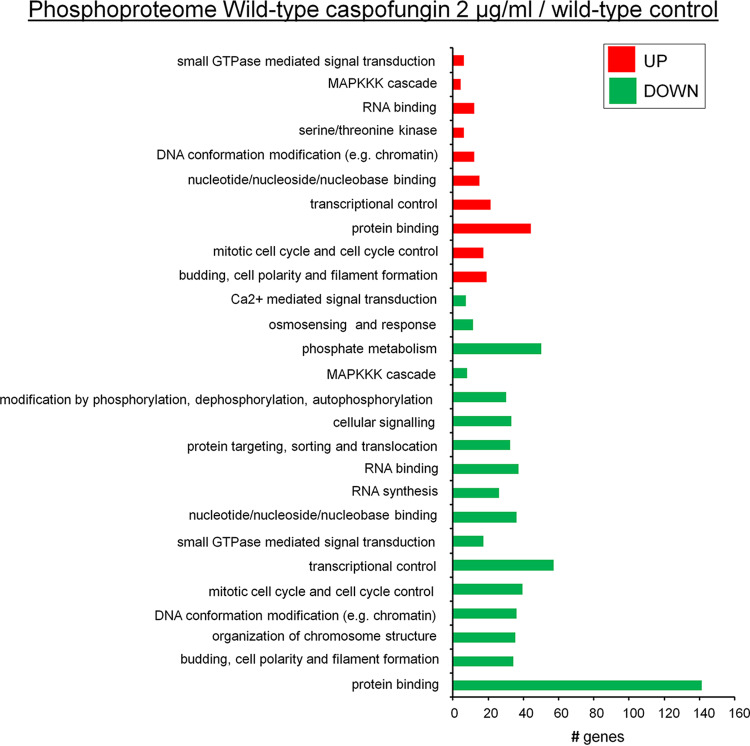
Functional categorization of phosphopeptides differentially phosphorylated in A. fumigatus wild-type strain exposed to 2 μg/ml caspofungin treatment for 1 h (*P* < 0.05). The functional enrichment was performed with FungiFun (https://sbi.hki-jena.de/fungifun/fungifun.php), using A. fumigatus Af293 as the organism and FunCat as the ontology classification.

A functional categorization was performed for proteins and phosphopeptides modulated in the wild-type strain during the drug response. Most of the proteins were found to have unknown functions, but four of them were identified as related to the deoxyribonucleotide metabolism (Fig. 1D). A total of 6,030 phosphopeptides modulated in the wild-type response have known function (Fig. 1E). Results of functional categorization of those phosphopeptides are shown in Fig. 2. Functional characterization of proteins that were downregulated and upregulated with respect to phosphorylation in the presence of caspofungin showed high complexity (Fig. 2). Enrichment was seen for proteins that were downregulated and upregulated with respect to phosphorylation and involved in small-GTPase-mediated signal transduction; the MAPK kinase kinase kinase (MAPKKK) cascade; the mitotic cell cycle; cell cycle control, cell budding, and cell polarity; filament formation; and transcriptional control (Fig. 2). Interestingly, MAPKKK cascade proteins such as BckA^Bck1^, SskB^Ssk2^, SteC^Ste11^, MpkA^Slt2^, and SakA^Hog1^ and the MAP phosphatase PtcG (26) were found to be downregulated or upregulated or both (see Table S1 at https://doi.org/10.6084/m9.figshare.12315212).

### Analysis of the Δ*mpkA and* Δ*sakA* proteome under the control of the fungal response to caspofungin stress.

**FIG 3:**
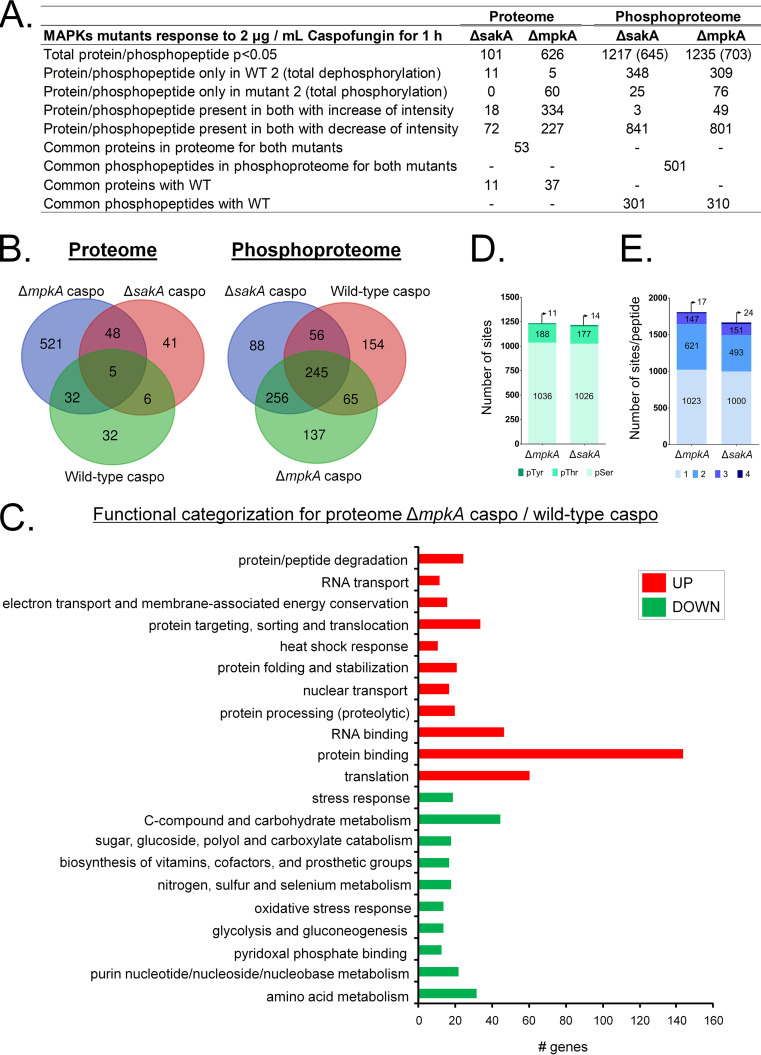
A. fumigatus Δ*mpkA* and Δ*sakA* proteomic and phosphoproteomic analysis. (A) Summary table of all proteins and phosphopeptides with difference in intensity between mutant samples (Δ*mpkA* and Δ*sakA* strains) and the wild-type strain after 2 μg/ml caspofungin treatment for 1 h (*P* < 0.05). (B) Venn diagram of proteins (proteomes) and phosphopeptides with difference in intensity between mutant (Δ*mpkA* caspo and Δ*sakA* caspo) samples and WT samples and between wild-type samples after 2 μg/ml caspofungin treatment for 1 h and WT samples at time zero (T0) (*P* < 0.05). (C) Functional enrichment was performed with FungiFun (https://sbi.hki-jena.de/fungifun/fungifun.php), using A. fumigatus Af293 as the organism and FunCat as the ontology classification. (D) Number of phosphorylated residues of serine, threonine, or tyrosine identified after phosphoproteomic analysis for both mutant strains. (E) Number of sites of phosphorylation per peptide for both mutant strains.

There is an involvement of MAPKs in the response to caspofungin and the modulation of the CPE (13, 15–17, 27, 28). The study of the global profile of protein levels and of protein phosphorylation in strains deleted for those kinases may provide the identification of new targets related to MAPK signaling pathways and drug responses. Results of a total comparison between proteome and phosphoproteome data from the wild-type (WT caspo/control), Δ*sakA* (Δ*sakA* caspo/WT caspo), and Δ*mpkA* (Δ*mpkA* caspo/WT caspo) strains are shown in Fig. 3A (see also Tables S2 and S3 at https://doi.org/10.6084/m9.figshare.12315212). The Δ*mpkA* strain was found to have the most distinct proteome profile, with a high number of unique proteins modulated, in comparison with WT and Δ*sakA* strains (Fig. 3A).

The results of proteomic study of the Δ*sakA* mutant strain showed that 11 proteins were not present in Δ*sakA* samples (i.e., were exclusively produced by the WT strains under the same treatment conditions), that 72 proteins had decreased intensity in mutant samples, and that 18 proteins had increased total levels (Fig. 3A and B; see also Table S3A at https://doi.org/10.6084/m9.figshare.12315212). As shown by Δ*mpkA* proteomic data representing results from treatment with caspofungin at 2 μg/ml, 5 proteins were exclusively produced by the WT samples, and 227 proteins showed a decrease of total level in the mutant strain. Most of the proteins (334) showed increased levels, and 60 proteins were present only in the mutant strain (not found in WT samples) (Fig. 3A and B; see also Table S3A).

In the proteome analysis of the Δ*mpkA* mutant strain, the functional enrichment data showed upregulation of proteins involved in electron transport and membrane-associated energy conservation, heat shock proteins, and translational proteins, as previously described (24) (Fig. 3C; see also Table S2B at https://doi.org/10.6084/m9.figshare.12315212). For the Δ*sakA* proteome, the results showed enrichment in downregulated proteins involved in glycolysis and gluconeogenesis (7 proteins) and C-compound and carbohydrate metabolism (23 proteins) (see Table S3A).

These data indicate that control of proteins involved in metabolism, such as in production of secondary metabolites, was highly represented in both mutants, suggesting that both kinases were under the control of production of metabolites as a response to caspofungin stress.

### Analysis of Δ*mpkA* and Δ*sakA* phosphoproteome under the control of the fungal response to caspofungin stress.

Results of phosphoproteomic analysis of mutants versus wild-type strain indicated a total of 1,217 and 1,235 phosphopeptides showing statistically significant differences in comparisons of the Δ*mpkA* mutant to the WT strain (Δ*mpkA*/WT) or Δ*sakA*/WT, respectively, during 1 h of incubation with 2 μg/ml caspofungin (Fig. 3A and B; see also Tables S2B and S3B at https://doi.org/10.6084/m9.figshare.12315212). A total of 245 phosphoproteins were found to be shared among the wild-type, Δ*mpkA*, and Δ*sakA* strains (Fig. 3B; see also Tables S1, S2B, and S3B). A total of 137 phosphoproteins were unique to the Δ*mpkA* mutant, while 88 were unique to the Δ*sakA* mutant (Fig. 3B; see also Tables S2B and S3B). There was a predominant decrease in phosphorylation seen for both mutants in comparison with the WT strain. Most (>75%) of the phosphosites identified were serine residues followed by threonine residues and a minimal percentage of tyrosine residues (Fig. 3C). Most of the phosphopeptides were found to have one phosphorylation site per peptide, but four phosphorylation sites per peptide were observed in a few cases (Fig. 3D).

**FIG 4:**
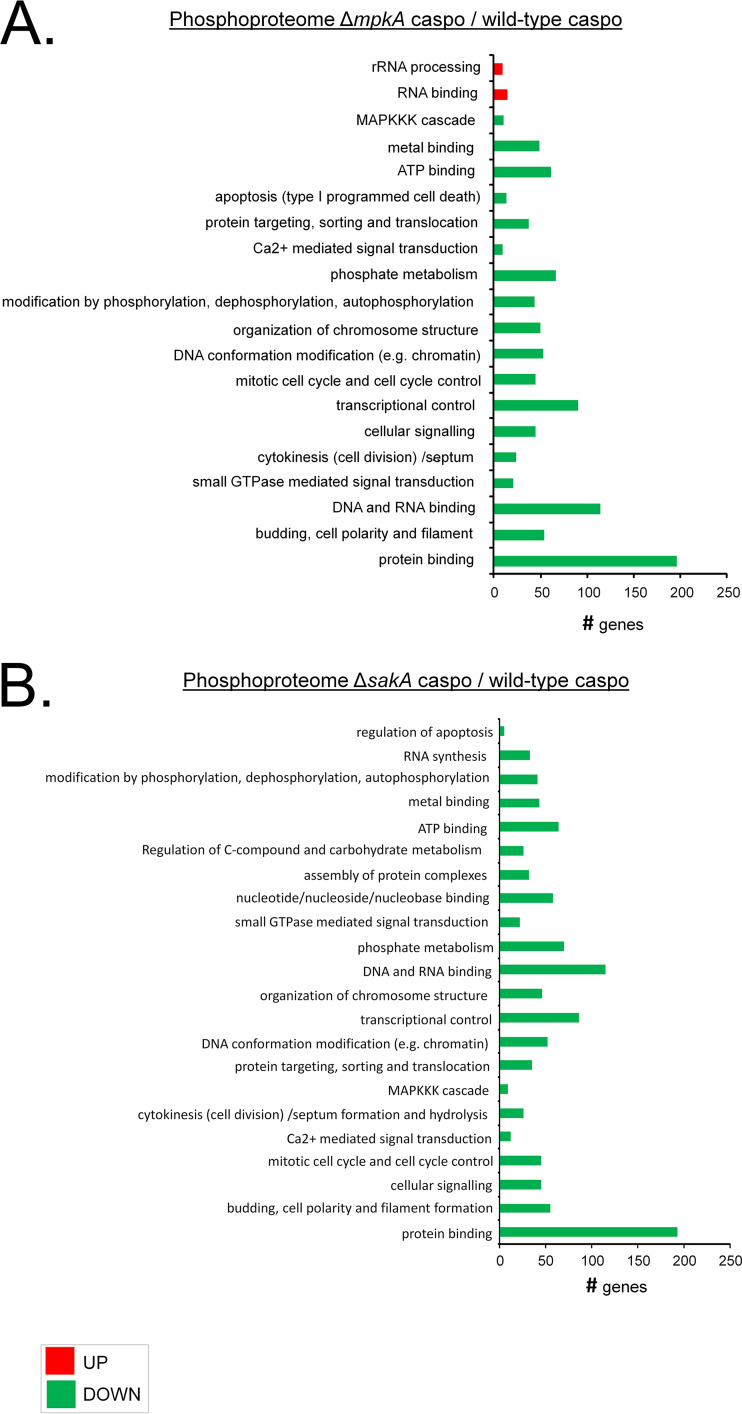
Phosphoproteome functional categorization of A. fumigatus Δ*mpkA* and Δ*sakA* mutants. Functional categorization of phosphopeptides differentially phosphorylated in the Δ*mpkA* strain (*P* < 0.05) (A) or Δ*sakA* strain (*P* < 0.05) (B) was performed. Functional enrichment was performed with FungiFun (https://sbi.hki-jena.de/fungifun/fungifun.php), using A. fumigatus Af293 as the organism and FunCat as the ontology classification.

The results of the functional categorization of phosphopeptides from both mutants were very similar and showed a high number of proteins with decreased phosphorylation involved in budding, cell polarity, and filament formation; Ca^2+^-mediated signal transduction; cellular signaling; cytokinesis (cell division)/septum formation and hydrolysis; the MAPKKK cascade; the mitotic cell cycle; cell cycle control; modification by phosphorylation, dephosphorylation, and autophosphorylation; small-GTPase-mediated signal transduction; and transcriptional control (Fig. 4; see also Table S2B and C). In addition, the Δ*sakA* mutant showed decreased phosphorylation of proteins involved in nucleotide/nucleoside/nucleobase binding, regulation of C-compound and carbohydrate metabolism, and RNA synthesis (Fig. 4B; see also Table S2B at https://doi.org/10.6084/m9.figshare.12315212).

Taken together, these data strongly suggest that MpkA and SakA collaborate in orchestrating the global phosphorylation of several proteins involved in the response to caspofungin.

### Kinases and transcription factors are involved in the fungal response to caspofungin.

Our major interest is in the identification of kinases and transcription factors that may be regulated in A. fumigatus during caspofungin responses, which might be under the control of fungal MAPKs. There are 28 kinases modulated in the wild-type strain (Tables 1 and 2). A total of 44 kinases were identified in WT, Δ*mpkA*, and Δ*sakA* samples treated with caspofungin (Table 1). Fourteen kinases were common for all of the strains, 12 kinases were common between the two null mutant strains, and 28 kinases were found to be modulated only during the WT response to caspofungin (Table 1). Table 2 shows the distribution of phosphopeptides in the MAP kinases from the three different MAP kinase modules in the wild-type, Δ*mpkA*, and Δ*sakA* strains upon exposure to caspofungin. All three MAP kinases from the cell wall integrity pathway were found to have phosphopeptides with modulated phosphorylation in the presence of caspofungin (Table 2). MAPKKK SskB and MAPK SakA in the HOG pathway and only the MAPKKK SteC in the invasive growth pathway were also found to have phosphopeptides with modulated phosphorylation (Table 2). Among the kinases exclusively associated with the WT strain, BckA (AFUA_3G11080) is an example of a protein with modulation of two different peptides, one with one site of increased phosphorylation (indicated in bold highlighting) (ESQAPSEGAPDT**S**PK) and a second one with two sites with dephosphorylation (indicated in bold highlighting) (SPRPQDD**S**DED**S**DDGLFAIPLSNNK) (Table 2; see also Table S1B at https://doi.org/10.6084/m9.figshare.12315212). The positions of the phosphopeptides indicate that S1039 and S1043 are closer to the active site of the kinase whereas S376 is closer to the N terminus of BckA. The S1039 residue can be phosphorylated by CKII, while the S1043 residue can be a substrate of CKI and CKI I (see Table S1B).

**TABLE 1:** Protein kinases with modulation of phosphorylation

Gene ID	Gene name	Protein name	Modulation^*a*^		
			WT caspo/ control	Δ*mpkA* caspo/ WT caspo	Δ*sakA* caspo/ WT caspo
AFUA_3G12670	*pkh2*^*b*^	Serine/threonine protein kinase, putative	**↓↑**	↓	↓
AFUA_6G06870	*yck2*^*b*^	Casein kinase I homolog, putative	↑	↓	↓
AFUA_5G03160	*ctk1*^*b*^	Protein kinase, putative	↓	↓↓	↓↓
AFUA_1G09950	*cbkA*	Casein kinase II subunit beta (CK II beta)	↓	↓	↓
AFUA_5G11520	*nrc2*	Serine/threonine protein kinase (Nrc-2), putative (EC 2.7.1.-)	↓	↓	↓
AFUA_5G05980	*tos3*^*b*^	Calcium/calmodulin dependent protein kinase, putative	↓↓	↓	↓
AFUA_1G14810	*kin4*	Serine/threonine protein kinase (Kin4), putative (EC 2.7.11.1)	↓	↓	↓
AFUA_2G12210	*sin1*	Stress-activated MAP kinase interacting protein, putative	↓	↓	↓
AFUA_3G10000	*pkaR*	cAMP-dependent protein kinase regulatory subunit (PKA regulatory subunit)	↓	↓	↓↓↓
AFUA_2G01700	*snf1*^*b*^	Nonspecific serine/threonine protein kinase (EC 2.7.11.1)	↓	↓↓↓↓	↓↓↓↓
AFUA_5G11840	*hrk1*^*b*^	Protein kinase, putative (EC 2.7.1.-)	↓	↓	↓↓
AFUA_2G15010	*srrB*^*c*^	Serine threonine protein kinase, putative	↓	↓↓↓	↓↓
AFUA_1G11080	*kin1*	Nonspecific serine/threonine protein kinase (EC 2.7.11.1)	↓↓↓↓	↓	↓
AFUA_6G08120	*sldA*	Checkpoint protein kinase (SldA), putative	↓	↓	↓
AFUA_2G14200	*prr1*^*b*^	Protein kinase, putative	−	↓↓	↓
AFUA_7G04330	*ste20*^*c*^	Ste20-like serine/threonine protein kinase, putative	−	↓	↓
AFUA_1G11930	*nnk1*^*b*^	Serine/threonine-protein kinase, putative	−	↓	↓
AFUA_3G08710	*isr1*^*c*^	Protein kinase domain-containing protein	−	↓	↓
AFUA_4G08920	*iks1*^*b*^	Protein kinase, putative	−	↓	↓
AFUA_4G06180	*ckb1*^*b*^	Casein kinase II subunit beta	−	↓	↓
AFUA_5G11970	*pkcA*	Protein kinase C	−	↓	↓
AFUA_6G04500	*gal83*^*b*^	Snf1 kinase complex beta-subunit Gal83, putative	−	↓↓	↓
AFUA_6G09240	*ypk2*^*b*^	Protein kinase	−	↓	↓
AFUA_1G16780	*lkh1*	Protein kinase (Lkh1), putative	−	↓	↓
AFUA_1G05800	*mkk2*	MAP kinase kinase (Mkk2), putative	−	↓	↓
AFUA_6G05120	*skp1*	Glycogen synthase kinase (Skp1), putative	↓	↓	−
AFUA_4G13720	*mpkA*	(MpkA) mitogen-activated protein kinase (EC 2.7.11.24)	↓↓	−	−
AFUA_2G13640	*kic1*^*b*^	Serine/threonin protein kinase, putative	↑	↓	−
AFUA_1G12940	*sakA*	(SakA) mitogen-activated protein kinase hog1 (MAP kinase hog1) (EC 2.7.11.24)	↑	−	−
AFUA_2G11730	*gin4*^*b*^	Protein kinase domain-containing protein	↓	−	−
AFUA_2G16620	*gcn2*^*b*^	Protein kinase, putative	↓	−	−
AFUA_4G01020	*fhk1*	Sensor histidine kinase/response regulator, putative (AFU_orthologue AFUA_4G01020)	↓	−	−
AFUA_7G03750	*cds1*	Serine/threonine-protein kinase chk2 (Cds1)	↓↓↓↓	−	−
AFUA_1G10940	*sskB*	MAP kinase kinase kinase (EC 2.7.11.-)	↑	−	−
AFUA_4G03140	*sky1*	Serine protein kinase Sky1, putative (EC 2.7.1.-)	↓↓	−	−
AFUA_1G06400	*schA*	Nonspecific serine/threonine protein kinase (EC 2.7.11.1)	↓	−	−
AFUA_5G06420	*steC*	MAP kinase kinase kinase SteC (EC 2.7.1.-)	↓	−	−
AFUA_3G11080	*bck1*	MAP kinase kinase kinase (Bck1), putative	↓↓↓	−	−
AFUA_2G01520	*yak1*^*b*^	Protein kinase, putative	↓	−	−
AFUA_2G09570	*stk-55*^*d*^	Serine/threonine protein kinase	−	↑	−
AFUA_2G10620	*ypk1*	Serine/threonine protein kinase (YPK1), putative	−	↓	−
AFUA_7G03720	*kin28*	Serine/threonine protein kinase (Kin28), putative	−	↑	−
AFUA_1G05930	*prk1*^*b*^	Serine/threonine protein kinase, putative	−	−	↓↓
AFUA_2G04680	*pakA*	Nonspecific serine/threonine protein kinase (EC 2.7.11.1)	−	−	↓

a The number of arrows represents the number of phosphopeptides identified for each protein (an arrow pointing down [↓] represents a phosphopeptide that has decreased phosphorylation whereas an arrow pointing up [↑] represents a phosphopeptide that has increased phosphorylation). −, the protein was not found under the described conditions.

b Gene name of orthologs in S. cerevisiae.

c Gene name of orthologs in A. nidulans.

d Gene name of orthologs in N. crassa.

**TABLE 2:** Phosphopeptides observed as differentially modulated in the presence of caspofungin in the mitogen-activated protein kinases^*a*^

Pathway and MAPK	WT caspo/ WT control	Δ*mpkA* caspo/ WT caspo	Δ*sakA* caspo/ WT caspo	Phosphopeptide
Cell wall integrity				
MAPKKK BckA (AFUA_3G11080)	Up (2.92)			364-ESQAPSEGAPDTSPK-378
		Down (0.28)		364-ESQAPSEGAPDTSPK-378
			Down (0.26)	364-ESQAPSEGAPDTSPK-378
		Down (0.08)		379-LSHEPQSAGPHSGTIENSPNLR-400
		Down		798-DAPQHTEGMSPVEGDQQVGISPEPDKADLLAR-829
			Down (0.26)	798-DAPQHTEGMSPVEGDQQVGISPEPDKADLLAR-829
	Down (0.47)			1032-SPRPQDDSDEDSDDGLFAIPLSNNK-1056
		(Down) 0.31		1032-SPRPQDDSDEDSDDGLFAIPLSNNK-1056
			Down	1032-SPRPQDDSDEDSDDGLFAIPLSNNK-1056
MAPKK Mkk2 (AFUA_1G05800)		(Down) 0.30		92-PAPPPLATTGLNESTGHSR-110
			Down	92-PAPPPLATTGLNESTGHSR-110
MAPK MpkA (AFUA_4G13720)	Down (0.12)			173-GFSIDPEENAGYMTEYVATR-192
	Down (0.16)			173-GFSIDPEENAGYMTEYVATR-192
		Up		173-GFSIDPEENAGYMTEYVATR-192

Invasive growth				
MAPKKK SteC (AFUA_5G06420)	Down			584-DSIASSSLQPLQEESPIEPNRK-605

High-osmolarity glycerol				
MAPKKK SskB (AFUA_1G10940)	Up			118-GSSVGAGAALDKVSPVDGLPLTDR-141
		Down (0.04)		118-GSSVGAGAALDKVSPVDGLPLTDR-141
MAPK SakA (AFUA_1G12940)	Up (1.8)			165-IQDPQMTGYVSTR-177

a “Down” and “Up” represent decreased and increased phosphorylation of a specific phosphopeptide, respectively. Numbers in parentheses represent fold change. MAPK, mitogen-activated protein kinase.

**FIG 5:**
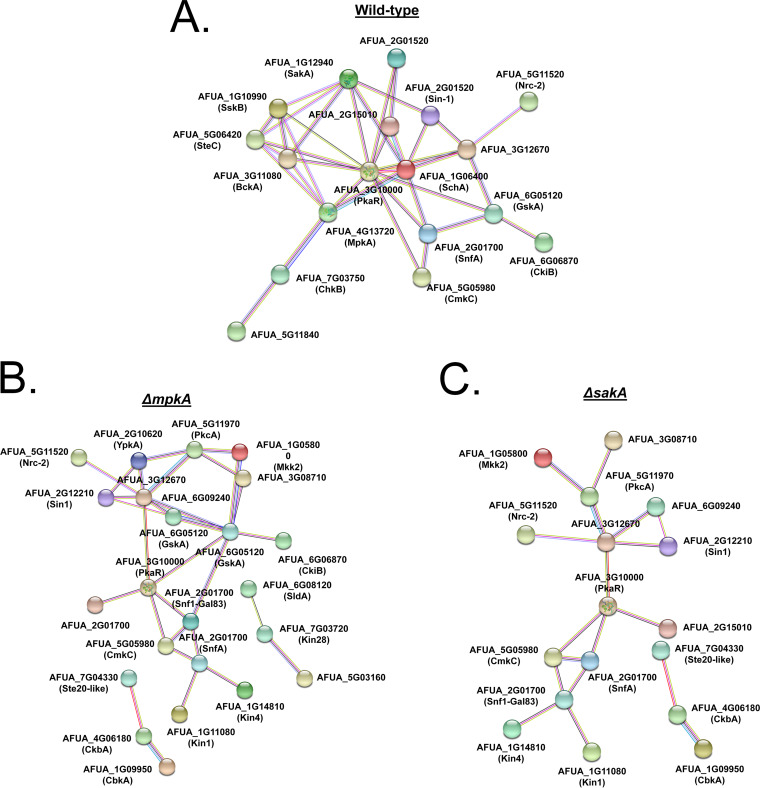
A. fumigatus protein kinase functional protein association network based on the protein phosphorylation profile during incubation with caspofungin. The sets of differentially protein kinase phosphorylated proteins from the wild-type (WT) (A), Δ*mpkA* (B), and Δ*sakA* (C) strains during caspofungin stress were combined for the generation of a general protein association network. Each edge represents a functional protein association retrieved from the STRING server (medium confidence threshold of 0.4 for the interaction score), and node sizes represent the degree of each node (number of edges connected to the node). Note that not all the differentially phosphorylated proteins are present in the network as many proteins did not present any functional associations within the whole set.

The phosphorylation profiles determined for kinases of the strains after exposure to caspofungin changed dramatically (Tables 1 and 2). In the wild-type strain, most of the kinases showed decreased phosphorylation compared to the untreated control, except for the mitogen-activated protein kinases SskB^Ssk2^ (AFUA_1G10940), SakA^Hog1^ (AFUA_1G12940), and Kic1 (AFUA_2G13640). There was also decreased phosphorylation seen in both the Δ*mpkA* mutant (except for AFUA_2G09570 and AFUA_7G03720, Kin28) and the Δ*sakA* mutant (Tables 1 and 2). To obtain insight into the integrated kinase signaling networks governing caspofungin phosphoproteomics, we generated functional gene networks using STRING analysis. The protein kinase interaction network generated for the wild-type strain showed not only the MAP kinase subnetwork (SakA^Hog1^, SskB^Ssk2^, SteC^Ste11^, BckA^Bck1^, and MpkA^Mpk1^) but also the cAMP-dependent protein kinases PkaR^Pka1^ and SchA^Sch9^ as highly connected (Fig. 5A). Although PkaR^Pka1^ was still present in the Δ*mpkA* and Δ*sakA* protein kinase interaction network, most of the MAP kinases and SchA^Sch9^, as well as the corresponding associated proteins, were not present (Fig. 5B and C). These results strongly indicate that MpkA and SakA have an important influence on global phosphorylation during caspofungin tolerance.

**FIG 6:**
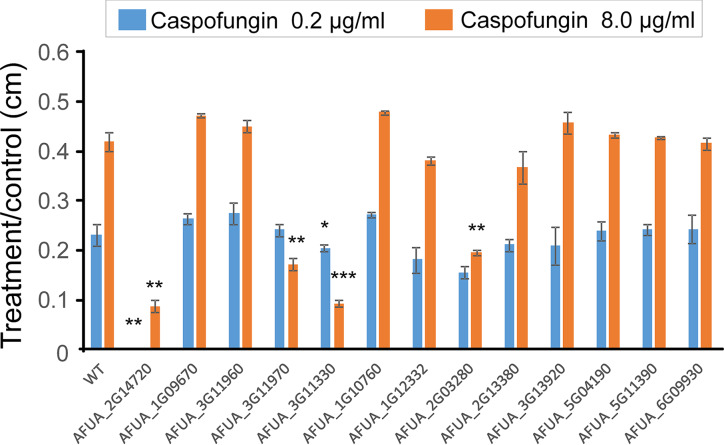
Analysis of A. fumigatus null TF strains grown on different concentrations of caspofungin. (A) fumigatus conidia (1 × 10^5^) were inoculated on solid minimal medium (MM) with different concentrations of caspofungin and grown for 5 days at 37°C. All plates were grown in triplicate, and averages ± standard deviations (SD) of data were plotted. A Student *t* test was performed using Prism GraphPad (version 6) to confirm the statistical significance of differences between treatment and control results (*, *P* < 0.05, **, *P* < 0.01; ***, *P* < 0.001).

There was also a great difference seen in the phosphorylation profiles generated for transcription factors (TFs) among strains postexposure to caspofungin (Table 3). In the wild-type strain, most of the TFs showed decreased phosphorylation compared to the untreated control, except for Sin3 (AFUA_8G05570). There was also decreased phosphorylation in both the Δ*mpkA* mutant (except for AFUA_6G09930, Yap1, and AFUA_3G13920, Mbp1) and the Δ*sakA* mutant (Table 3). As a first step in characterizing the influence of these TFs on caspofungin tolerance, we analyzed the ability of 13 null mutants of these TFs (29) to grow on caspofungin. We previously observed that a Δ*hapB* mutant (AFUA_2G14720, encoding a CAAT-binding TF) (30) and a Δ*atfA* mutant (AFUA_3G11330) were more susceptible to caspofungin at 0.2 μg/ml than the wild-type strain and showed reduced CPE (Fig. 6). A Δ*pacC* mutant (AFUA_3G11970, encoding a TF that undergoes activation in response to alkaline pH) (31), and a Δ*zipD* mutant (AFUA_2G03280, encoding a TF important for calcium metabolism and osmotic response) (9, 10), were previously found to have reduced CPE.

**TABLE 3:** Transcription factors with modulation of phosphorylation

Gene ID	Gene name	Protein name	Modulation^*a*^		
			WT caspo/ control	Δ*mpkA* caspo/ WT caspo	Δ*sakA* caspo/ WT caspo
AFUA_3G11970	*pacC*	C2H2 transcription factor PacC, putative	↓	↓	↓
AFUA_3G11330	*atfA*	BZIP transcription factor (AtfA), putative	↓	↓	↓
AFUA_1G09670	*glcD*	HLH transcription factor (GlcD gamma), putative	↓	↓	−
AFUA_3G02340	*ncb2*^*b*^	CBF/NF-Y family transcription factor, putative	↓	−	↓
AFUA_2G14720	*hapB*	CCAAT-binding transcription factor subunit HAPB	↓↓↓	−	↓↓↓
AFUA_2G03280	*zipD*	BZIP transcription factor, putative	−	↓↓	↓↓
AFUA_2G01900	*rtf1p*	RNA polymerase II transcription elongation factor Rtf1p, putative	−	↓	↓
AFUA_1G12332	*rph1*^*b*^	Jumonji family transcription factor, putative	−	↓	↓
AFUA_2G13380	*areB*	GATA transcription factor (AreB), putative	−	↓	↓↓
AFUA_3G11170	*csp-2*^*c*^	CP2 transcription factor, putative	−	↓↓↓↓	↓
AFUA_2G14250	*bur6*^*b*^	CBF/NF-Y family transcription factor, putative	−	↓	↓
AFUA_1G12260	*iws1*	Transcription factor iws1	−	↓	↓
AFUA_5G11390	*bqt4*^*d*^	APSES transcription factor, putative	−	↓↓↓↓↓	↓↓↓
AFUA_3G08520	*rlmA*	SRF-type transcription factor RlmA	−	↓	↓
AFUA_5G13310	*aro80*^*b*^	C6 transcription factor, putative	−	↓	↓
AFUA_8G05570	*sin3*	Transcription factor (Sin3), putative	↑	−	−
AFUA_1G10760	*mak21*^*b*^	CCAAT-box-binding transcription factor	↓↓	−	−
AFUA_7G04710	*fap1*^*b*^	NF-X1 finger transcription factor, putative	↓	−	−
AFUA_3G11960	*fkh2*^*b*^	Forkhead transcription factor Fkh1/2, putative	↓↑	−	−
AFUA_1G06900	*crzA*	C2H2 finger domain transcription factor CrzA	↓	−	−
AFUA_2G17220	*amdX*	C2H2 transcription factor (AmdX), putative	↓	−	−
AFUA_2G14800	*hpa3*	HLH transcription factor (HpaIII), putative	↓	−	−
AFUA_5G03430	*rum1*	PHD transcription factor (Rum1), putative	↓	−	−
AFUA_6G09930	*yap1*	BZIP transcription factor AP-1/Yap1, putative	−	↑	−
AFUA_3G13920	*mbp1*^*b*^	APSES transcription factor, putative	−	↓↓	−
AFUA_7G05620	*mpbA*	APSES transcription factor (MbpA), putative	−	↑	−
AFUA_5G04190	*palcA*	HLH transcription factor (PalcA), putative	−	−	↓

a The number of arrows represents the number of phosphopeptides identified for each protein (an arrow pointing down [↓] represents a phosphopeptide that has decreased phosphorylation whereas an arrow pointing up [↑] represents a phosphopeptide that has increased phosphorylation). −, the protein was not found under the described conditions.

b Gene name of ortholog in S. cerevisiae.

c Gene name of ortholog in N. crassa.

d Gene name of ortholog in S. pombe.

## DISCUSSION

Echinocandins, including caspofungin, anidulafungin, and micafungin, represent the second line of treatment for invasive aspergillosis (32, 33). Echinocandins act by the inhibition of the protein Fks1, a component of fungal membrane, resulting in the disruption of the synthesis of the polysaccharide β-(1,3) glucan, a major component of the fungal cell wall (33). In this work, we performed proteome and phosphoproteome analyses of a WT strain and of MAPK null mutants (the *ΔmpkA* and *ΔsakA* strains) under conditions of high caspofungin concentrations in liquid medium, aiming to identify new targets related to the CPE in A. fumigatus. For the analysis of wild-type responses to caspofungin, we identified 814 phosphopeptides (520 unique proteins) with modulation of phosphorylation. Most of the phosphopeptides suffered a decrease of phosphorylation, with a predominance of proteins being related to transcriptional control. The analysis of the response of the Δ*mpkA* strain to caspofungin indicates that 1,235 phosphopeptides were being modulated (703 unique proteins), most of them with decreased phosphorylation, such as was observed for the WT response to caspofungin. The modulation of proteins involved in transcriptional control and phosphate metabolism was predominant, as was also observed for the Δ*sakA* phosphoproteome.

The participation of MpkA and SakA in the caspofungin response is well established (6, 9, 16, 27). Activation of CWI pathways, together with increased MpkA phosphorylation, occurs at lower concentrations of caspofungin (9). However, under conditions of high concentrations, the phosphorylation of the kinase is reduced (9). In addition, it is known that chitin biosynthesis in Candida albicans can be modulated by components of the high-osmolarity glycerol (HOG) pathway (7). It has also been demonstrated that, in A. fumigatus treated with subinhibitory concentrations of caspofungin, an *in silico* interaction may occur between SakA and MpkA (16). Here, we showed the involvement of MpkA and SakA in the modulation of phosphorylation of different targets during the response to high doses of caspofungin. We observed that MpkA and SakA suffered dephosphorylation and phosphorylation, respectively, when the wild-type strain was exposed to caspofungin.

Modulation of protein kinases and transcription factors in the response to caspofungin stress was highly represented in all analyses performed. The protein kinase A (PKA) regulatory subunit (AFUA_3G10000), for example, showed decreased phosphorylation (site indicated in bold highlighting) in the wild-type strain exposed to caspofungin (site RT**S**VS, ratio = 0.5), which was also observed in *ΔmpkA* and *ΔsakA* phosphoproteomes. In the MAPK null mutant strains, four sites of phosphorylation (indicated with bold highlighting) were identified with dephosphorylation (for the *ΔsakA* phosphoproteome, sites KY**S**PI [ratio = 0], RT**S**VS [ratio = 0.12] VT**S**PT [ratio = 0.13], and P**S**PS [ratio = 0.28]; for the *ΔsakA* phosphoproteome, sites P**S**PS [ratio = 0], KY**S**PI [ratio = 0], VT**S**PT [ratio = 0.03], and RT**S**VS [ratio = 0.06]). There is a physical interaction between A. fumigatus SakA and MpkC (17, 34) and the PKA regulatory subunit (35). Carbohydrate mobilization is controlled by SakA interactions with PkaC1 catalytic and PkaR regulatory subunits, suggesting a putative mechanism where the PkaR regulatory subunit leaves the complex and releases the SakA-PkaC1 complex for activation of enzymes involved in carbohydrate mobilization (35). These results suggest a possible participation of PKA, CWI, and HOG pathways in the mobilization of carbohydrate for cell wall remodeling and for responses to caspofungin. In addition, the importance of PKA in fungal viability was also observed in strains deleted for *pkaC1*, which suffered defects in germination and in cell wall organization (36–38). Here, protein kinase C (PKC; AFUA_5G11970) was also identified with dephosphorylation in both the Δ*mpkA* and Δ*sakA* mutant strains. PKC acts in the CWI pathway in Saccharomyces cerevisiae and A. fumigatus by the activation of MAPKs with involvement of Bck1 (a MAPKKK, dephosphorylated in WT caspofungin samples), Mkk1/Mkk2 (Mkk2 [AFUA_1G05800], with dephosphorylation in both null mutant strains), and MpkA (AFUA_4G13720, with dephosphorylation only in the WT) (39–43).

Some transcription factors identified here have already been shown to be related to CPE in A. fumigatus, such as AtfA (AFUA_3G11330), a homologue of ATF1 (which also includes AtfB or AtfD) (14), with dephosphorylation in the WT, *ΔmpkA*, and *ΔsakA* strains. The ZipD transcription factor (AFUA_2G03280), with dephosphorylation in both mutant strains, also plays a role in the caspofungin-induced CWI response pathway (9, 10). AreB (AFUA_2G13380), with a decrease of phosphorylation in both null mutant strains, was also identified with a modulation of phosphorylation in A. fumigatus responses to Congo red (25).

In our study, we demonstrated the complexity of the fungal response to caspofungin stress. The members of the main group of proteins with strong modulation of phosphorylation are related to the transcription control, followed by proteins related to phosphate metabolism and transfer, in which kinases were highly represented. However, the modulation of proteins related to cytoskeletal organization and heat shock responses and β-1,3-glucan synthase is also important; in addition to calcium-calcineurin pathway and CWI pathways, all of them have already been described in the literature as important for the CPE in A. fumigatus and other fungi (7, 8, 10, 19, 28, 44, 45).

Increased understanding of how the modulation of protein phosphorylation may affect the fungal growth in the presence of caspofungin represents an important step in the development of new strategies and methods to combat the fungus inside the host. We showed that the phosphorylation profile is strongly modulated during the drug response, which can be also related to groups of proteins that have already been identified and described as showing level changes during the CPE response (28, 46). We have also shown that the MAPKs MpkA and SakA may control the CPE response by the modulation of the phosphorylation of new targets, which deserves further investigation by future studies.

## MATERIALS AND METHODS

### Fungal growth, protein extraction, digestion, and phosphoenrichment.

Aspergillus fumigatus conidia (1 × 10^7^) from the wild-type (WT) strain and mutant strains (Δ*mpkA* and Δ*sakA* mutants) (strain details are shown in Table S11 at https://doi.org/10.6084/m9.figshare.12315212) were inoculated into YG medium {0.5% yeast extract, 1% dextrose, 0.1% trace elements [22.0 g/liter ZnSO_4_, 11 g/liter boric acid, 5 g/liter MnCl_2_, 5 g/liter FeSO_4_, 1.6 g/liter CoCl_2_, 1.6 g/liter CuSO_4_, 1.1 g/liter (NH4)_2_MoO_4_]} and incubated for 16 h with rotation at 200 rpm at 37°C. After growth of mycelia, 2 μg/ml of caspofungin was added to the flasks, followed by incubation for 1 h. Mycelia were filtered using a vacuum system, harvested, and frozen by the use of liquid nitrogen. Frozen mycelia were macerated, resuspended in 1 ml TNE buffer (50 mM Tris-HCl [pH 7.5], 140 mM NaCl, 5 mM EDTA, EDTA-free protease inhibitor cocktail [Roche], 0.1 mM phenylmethylsulfonyl fluoride [PMSF], 100 mM NaF, 1 mM Na_3_VO_4_, 0.05 mM sodium β-glycerophosphate) and incubated for 15 min with agitation following centrifugation at 13,000 × *g* for 10 min. The supernatant was collected and total protein quantified Bradford assay (47). A 500-μg volume of protein was precipitated by addition of trichloroacetic acid (TCA) at a final 10% (wt/vol) concentration, subjected to vortex mixing for 15 s, and placed on ice for a minimum of 30 min. Samples were centrifuged at 14,000 × *g* for 15 min, and the supernatant was discarded. The pellet was washed three times with cold acetone with centrifugation at 14,000 × *g* (4°C) for 10 min. Proteins were dissolved in 150 μl of 8 M urea and 50 mM ammonium bicarbonate (Ambic). Dithiothreitol (DTT) (10 mM) was added followed by incubation for 45 min at 30°C. Protein alkylation was performed by addition of 40 mM iodoacetamide and incubation for 30 min at room temperature in the dark; 5 mM DTT was added with 15 min of incubation at 30°C. Ambic (50 mM) was added for urea dilution, and trypsin was added at a trypsin/protein ratio of 1:50. Protein digestion was performed by an overnight (16-h) incubation at 30°C. The digestion was stopped by addition of trifluoroacetic acid (TFA) to reach a final concentration of 1%. Samples were desalted by the use of an Oasis MCX Plus short cartridge (Waters), and peptide concentrations were determined by the use of a Qubit protein assay kit (Thermo Fisher Scientific). From the total peptides, 5% was collected for proteomic analysis, and 95% was used for phosphopeptide enrichment with TiO_2_ resin (Titansphere; GL Sciences Inc., Japan) in batch mode. Samples were resuspended in 80% acetonitrile (ACN)–1 M glycolic acid–5% TFA and mixed into the resin (1 mg resin to 500 μg peptide) and incubated for 20 min at room temperature. Resin was washed three times with 80% ACN–1% TFA, and phosphopeptides were eluted with 0.5% NH_4_OH (48, 49).

### Nano-LC-MS/MS (nano-liquid chromatography–tandem mass spectrometry) analysis.

Peptide samples were resuspended in 0.1% formic acid (FA) before analysis using a nano-flow EASY-nLC 1200 system (Thermo Scientific) coupled to an Orbitrap Fusion Tribrid mass spectrometer (Thermo Scientific) (Instituto de Química, Universidade de São Paulo). The peptides were loaded on an Acclaim PepMap C_18_ (Thermo Scientific, Germany) trap column (2 cm in length, 100-μm inner diameter; 5-μm pore size) and separated onto an Acclaim PepMap C_18_ (15 cm in length, 75-μm inner diameter; 3-μm pore size) column and separated with a gradient from 100% mobile phase A (0.1% FA) to 28% phase B (0.1% FA, 80% ACN) for 70 min, 28% to 40% for 10 min, and 40% to 95% for 2 min and 12 min at 95% at a constant flow rate of 300 nl/min. The mass spectrometer was operated in positive-ion mode with data-dependent acquisition. The full scan was acquired in the Orbitrap instrument at a resolution of 120,000 FWHM (full width at half-maximum) in the 375 to 1,600 *m*/*z* mass range with a maximum injection time of 50 ms and an automatic gain control (AGC) target of 5E−5. Peptide ions were selected using the quadrupole with an isolation window of 1.2 and were fragmented with high-energy collisional dissociation (HCD) MS/MS using a normalized collision energy value of 35 and were detected in the ion trap. Data-dependent acquisition performed with a cycle time of 3 s was used to select the precursor ions for fragmentation. Dynamic exclusion was activated with 12 s as exclusion duration and 20 ppm as the mass tolerance. All raw data were accessed in Xcalibur software (Thermo Scientific).

### Database searches and bioinformatics analyses.

Raw data were processed using MaxQuant software version 1.5.2.8 and the embedded database search engine Andromeda. The MS/MS spectra were searched against the UniProt Aspergillus fumigatus Protein Database (downloaded October 2017; 9,648 entries), with the addition of common contaminants, with accuracies of 4.5 ppm for MS and 0.5 Da for MS/MS. Cysteine carbamidomethylation (57.021 Da) was set as the fixed modification, with two missed cleavages for trypsin. Methionine oxidation (15.994 Da), protein N-terminal acetylation (42.010 Da), and phosphorylation S/T/Y (+79.96 Da) were set as variable modifications. Proteins and peptides were accepted at a false-discovery rate (FDR) of less than 1%. Label-free quantification was performed using MaxQuant software with the “match between run” and iBAQ features activated. The levels of MS intensity of phosphopeptides determined under the different conditions were compared, and the Student *t* test was used for the comparisons. Three biological replicates for each strain were analyzed. Plots of results of principal-coordinate analysis (PCA) comparing all untreated replicates (control) and caspofungin (2 μg/ml)-treated replicates, for proteome or phosphoprotrome samples, are indicated in Fig. S1 (available at https://doi.org/10.6084/m9.figshare.12315212).

The functional enrichment was performed with FungiFun (https://sbi.hki-jena.de/fungifun/fungifun.php), using A. fumigatus Af293 as the organism, FunCat as the ontology classification, and identifiers (IDs) from each supplemental table (only “AFUA_” IDs were used).The protein-protein interaction networks were obtained by the use of STRING (https://string-db.org/) and Cytoscape version 3.6.1.
